# Perinatal outcomes of intrahepatic cholestasis during pregnancy: An 8-year case-control study

**DOI:** 10.1371/journal.pone.0228213

**Published:** 2020-02-19

**Authors:** Chloé Arthuis, Caroline Diguisto, Henri Lorphelin, Vincent Dochez, Emmanuel Simon, Franck Perrotin, Norbert Winer

**Affiliations:** 1 Department of Gynecology and Obstetrics, University Hospital Regional Center Tours, Tours, France; 2 Department of Gynecology and Obstetrics, University Hospital Center Nantes, Nantes, France; Texas A&M University, UNITED STATES

## Abstract

**Introduction:**

Previous studies of fetal effects have suggested that intrahepatic cholestasis of pregnancy is associated with a higher rate of adverse neonatal outcomes including preterm birth, neonatal respiratory distress syndrome, meconium-stained amniotic fluid, neonatal intensive care unit admission, and stillbirth. The objective was to compare the neonatal and maternal consequences in pregnancies affected by intrahepatic cholestasis and normal pregnancies.

**Material and methods:**

This case-control study compares pregnancies affected by intrahepatic cholestasis (pruritus and bile acid ≥ 10 μmol/L) with low-risk pregnancies managed between December 2006 and December 2014 at a French university hospital center.

**Results:**

There were 83 (59.3%) cases of mild cholestasis (10≤ BA ≤39 μmol/L), 46 (32.8%) of moderate cholestasis (40≤ BA ≤99 μmol/L), and 11 (7.9%) of severe cholestasis (BA ≥100 μmol/L). No in utero fetal deaths occurred in the 140 women with cholestasis or the 560 controls analyzed. The rate of respiratory distress syndrome was higher in neonates of women with intrahepatic cholestasis (17.1% vs. 4.6%, *P*<0.001; crude OR 4.46 (CI95% 2.49–8.03)). This risk was also significant after adjustment for gestational age at birth and mode of delivery, adjusted OR 2.56 (CI95%1.26–5.18). The postpartum hemorrhage rate was twice as high among the case mothers (25% versus 14.1% for controls, *P* = 0.002).

**Conclusion:**

After adjustment on the confounding factors we found a higher rate of respiratory distress syndrome and neonatal morbidity among neonates of the cholestasis group.

## Introduction

Intrahepatic cholestasis of pregnancy (ICP) is the most common liver disease during pregnancy, occurring most often during its second and third trimesters. Its prevalence is estimated at 0.5% in France and varies with ethnicity. ICP is defined by pruritus *sine materia* (without lesions), with elevated levels of serum bile acids (BA) and liver enzymes. Its pathogenesis is still unknown, but hormonal influence and the MDR3 mutation gene (multidrug resistance 3) may contribute to it. Symptoms and abnormal liver function are spontaneously reversible after delivery, with good maternal prognosis. Moreover, maternal treatment with ursodeoxycholic acid improves pruritus and laboratory abnormalities and extends pregnancy [1]. Previous studies of fetal effects have suggested that ICP is associated with a higher rate of adverse neonatal outcomes including preterm birth, neonatal respiratory distress syndrome (RDS) [2], meconium-stained amniotic fluid [3], neonatal intensive care unit admission, and stillbirth. The prevalence of in utero and perinatal mortality is estimated at 0.5%. Severe cholestasis with higher BA levels is associated with a higher risk of fetal complications [1–2]. The mechanisms relating cholestasis to stillbirth remain uncertain: in utero deaths imputable to cholestasis occur during pregnancies with other pathologies [4]. Several experimental animal studies have shown that high BA levels have a harmful effect on cardiomyocytes [5]. Thus, it has been hypothesized that ICP might induce fetal arrhythmia that may lead to stillbirth. Perez et al. reported no deleterious effects of an acute high dose of cholic acid administered to a pregnant ewe, which suggests that the harm might require exposure over some period of time [6].

Thus far, both prenatal management and optimal time to delivery remain unclear. No method of fetal monitoring has been shown to either predict adverse perinatal outcomes or reduce their risk. The recommendations of various national professional societies for time to delivery in ICP-complicated pregnancies are also divergent. The Royal College of Obstetrics and Gynaecology does not endorse routine early delivery of these pregnancies [7], while the American College of Obstetricians and Gynecologists supports active management induction of labor protocols for ICP [8].

Our level III reference center, like most French maternity units, uses active management of ICP, defined by weekly clinical and laboratory monitoring with systematic induction of labor before or by 38 weeks of gestation. The exact term depends on its severity. This active attitude aims to avoid stillbirth [9], although its imputability to cholestasis has not been clearly established [10,11]. This study aimed to evaluate neonatal and maternal outcomes of this routine induction in the ICP cases, compared with controls.

## Materials and methods

### Patient selection and data collection

This case-control study included 140 women identified with ICP and 560 controls from December 2006 to December 2014. Cholestasis was diagnosed by the association of pruritus and BA ≥10 μmol/L (after other causes of itching and liver dysfunction were ruled out), and by normalization of biochemical parameters after delivery. Recurrences of cholestasis (each woman was included only one time) over the study period, multiple pregnancies, congenital malformations, and chromosomal abnormalities were excluded. Eligible control women had a singleton in cephalic presentation, without congenital malformations and no obstetric disorders requiring preterm induction. The control population was matched for maternal age (one year more or less), date of delivery (same calendar year), same parity, and did not have ICP. To improve the power study, four women with low-risk pregnancies were recruited as controls for each ICP case (ratio of 1:4; following 4 patients after each case).

We collected the following data for case and control women: demographic characteristics, pregnancy history, obstetric outcomes including term at delivery, spontaneous or induced labor, mode of delivery, meconium staining of amniotic fluid during labor, birth weight, postpartum hemorrhage (defined by blood loss ≥ 500 mL) and indicated transfusion. For neonatal status, adverse neonatal outcome was defined as pHa<7.10, an Apgar score <7 at 5 minutes, intubation, neonatal intensive care unit admission or perinatal death. For cases only, we also collected the history of liver diseases including viral hepatitis or calculous pancreatitis or cholecystitis; abnormal laboratory values at diagnosis, at delivery, and highest values during pregnancy (bile acids, transaminases, total bilirubin, hemoglobin, and hemostasis); treatment initiation; and clinical and laboratory course with treatment. BA was assayed weekly with an enzymatic spectrophotometric method (Olympus AU640 chemistry analyzer). Cholestasis severity was defined by the maximum BA rate rather than by BA level at diagnosis: mild if 10 ≤ BA ≤ 39 μmol/L, moderate if 40 ≤ BA ≤ 99 μmol/L, and severe if BA ≥100 μmol/L.

### Statistical analysis

Continuous data are presented as medians and interquartile ranges (1st quartile-3rd quartile) or means and their standard deviations, and categorical data as counts and percentages. Baseline characteristics, neonatal and maternal outcomes of cases and controls were compared. Mixed linear regression models were used to take the clustering effect of matching for quantitative data into account. Percentages were compared by conditional logistic regression. The cholestasis cases severity were compared by analysis of variance. The association between the composite neonatal outcome and ICP was estimated by crude and adjusted Odd ratios (adjustment on gestational age at birth and delivery mode). Analyses were conducted with R version 3.1.3. Differences were defined as significant when *P*<0.05.

### Ethical approval

The local ethics committee of university center of Tours approved this retrospective study. All data were fully anonymized before analyze in a secure database and ethics committee waived the requirement for informed consent.

## Results

**Fig 1:**
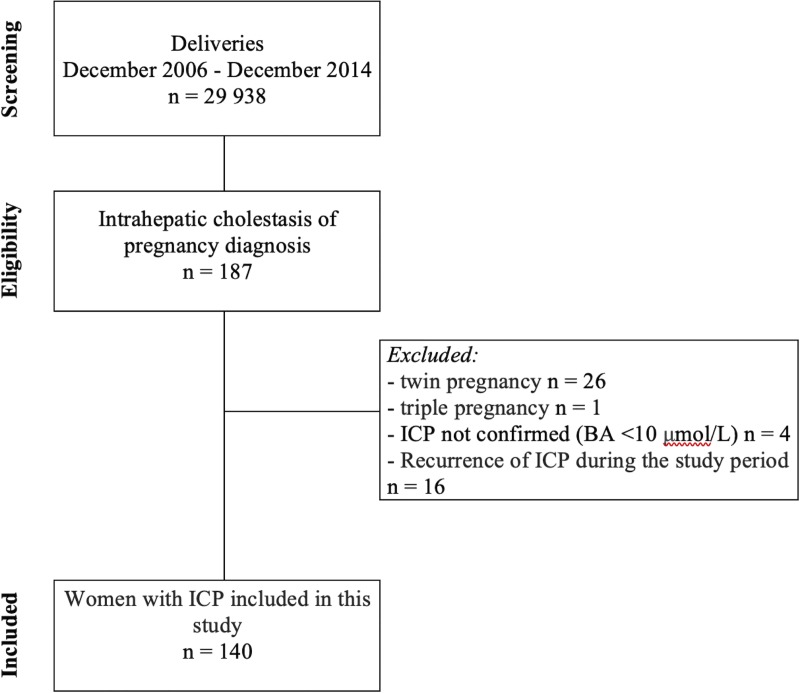
Flow chart.

Between December 2006 and December 2014, 29 938 women gave birth at the University Hospital Center. The study finally included 140 pregnancies with a confirmed diagnosis of ICP (Fig 1) for an incidence of 0.5%.

### Baseline characteristics

Table 1 summarizes the principal characteristics of the ICP group and the control group. They did not differ significantly for the matching criteria. Six women (4.3%) from ICP group had a personal history of liver disease: 5 women with chronic or treated hepatitis and 1 with autoimmune hepatitis.

**Table 1:** Maternal characteristics of women with intrahepatic cholestasis of pregnancy and the control group.

	Intrahepatic cholestasis of pregnancy (ICP) N = 140	Controls N = 560	P Values
Maternal age, years (median (IQR))	29 (25–34)	29 (25–34)	1
Nulliparous, n (%)	61 (43.6)	246 (43.9)	0.939
White, n (%)	118 (84.3)	435 (77.7)	0.086
BMI, kg/m2 (median (IQR))	23.6 (21–27.2)	22.4 (20–25.1)	0.216
Personal history of ICP, n (%)	31 (22.1)	1 (0.3)	<0.001
Family history of ICP, n (%)	4 (2.9)	0	<0.001
MDR3 gene mutation, n (%)	6 (4.3)	0	<0.001
Preexisting liver disease, n (%)	6 (4.3)	13 (2.3)	0.240
Preexisting coagulopathy, n (%)	5 (3.6)	18 (3.2)	0.957
Gestational diabetes, n (%)	22 (15.7)	25 (5.1)	<0.001
Hypertensive disorder, n (%)	2 (1.4)	3 (0.5)	0.574

### Neonatal outcomes

No stillbirth occurred during the 8-year study period in our population (Table 2). The mean birth weight was 3082 g (2825–3370 g) in the case population and 3350 g (3050–3610 g) (*P* <0.001) in the control population, which was the 50th percentile for term. The rate of small-for-gestational-age (SGA) fetuses did not differ between the groups, but neonatal status was worse in the cholestasis cases. Neonates exposed to cholestasis had a greater risk of having a RDS in comparison with controls (17.1% vs. 4.6%; *P*<0.001) crude OR 4.46 (95%CI: 2.49–8.03), even after adjustment on gestational age and delivery mode (aOR 2.56 (95%CI: 1.26–5.18)). The rates of admission to neonatal intensive care units was approximately three times higher in the cholestasis than the control group (*P* = 0.018). Moreover, neonatal morbidity was higher in cholestasis cases compared to controls OR 3.58 (95%CI: 2.03–6.27), and after adjustment aOR 2.28 (95%CI: 1.15–4.52) (Table 3).

**Table 2:** Neonatal and maternal outcomes of pregnancies with intrahepatic cholestasis of pregnancy (ICP).

	ICP N = 140	Controls N = 560	P Value
Obstetric outcomes			
Gestational age at birth, median (IQR)	38 (37–38)	40 (39–40)	<0.001
Preterm delivery before 37 weeks, n (%)	22 (15.7)	27 (4.8)	<0.001
Meconium-stained fluid, n (%)	26 (18.6)	121 (21.6)	0.501
Male, n (%)	74 (52.9)	282 (50.4)	0.664
Birth weight, grams, median (IQR)	3082 (2825–3370)	3350 (3050–3610)	<0.001
Small for gestational age, n (%)	8 (5.7)	18 (3.2)	0.161
Arterial pH, median (IQR)	7.29 (7.24–7.33)	7.27 (7.22–7.31)	0.199
Venous pH, median (IQR)	7.35 (7.30–7.38)	7.34 (7.29–7.37)	0.344
Arterial lactates, median (IQR)	3.5 (2.7–4.9)	4.3 (3.1–5.7)	0.060
Maternal outcomes			
Induction of labor, n (%)	115 (82.1)	103 (18.4)	<0.001
Type of delivery, n (%)			
Vaginal delivery	104 (74.3)	487 (86.9)	<0.001
Cesarean delivery during labor	19 (13.6)	61 (10.9)	0.457
Scheduled cesarean delivery	17 (12.1)	12 (2.1)	<0.001
Postpartum hemorrhage, n (%)	29 (20.7)	86 (15.35)	
Maternal transfusion, n (%)	4 (2.5)	6 (1.1)	0.232
Neonatal outcomes, n (%)			
5-min Apgar score <7, n (%)	4 (2.9)	3 (0.5)	0.046
Arterial pH<7.10, n (%)	4 (2.9)	8 (1.5)	0.423
Arterial pH<7.00, n (%)	0 (0)	2 (0.4)	0.999
Respiratory distress syndrome	24 (17.1)	26 (4.6)	<0.001
Mechanical ventilation or intubation	5 (3.6)	3 (0.5)	0.009
Admission to neonatal intensive care unit	4 (2.9)	2 (0.4)	0.018
Stillbirth	0 (0)	0 (0)	1

**Table 3:** Neonatal outcome according to antenatal exposition to cholestasis.

	ICP N = 140	Controls N = 560	Crude OR (CI95%)	aOR* (CI 95%)
Respiratory distress syndrome	N = 24/140 (17.1)	N = 26/560 (4.6)	4.46 (2.49–8.03)	2.56 (1.26–5.18)
Neonatal morbidity **	N = 25/140 (17.9)	N = 32/560 (5.7)	3.58 (2.03–6.27)	2.28 (1.15–4.52)

* OR adjusted for the following confounding factors gestational age at birth and mode of delivery.

** Neonatal morbidity was defined as Apgar score at 5 min less than 7, arterial cord blood pH less than 7.10, ventilation, intubation or external cardiac massage and admission to the neonatal intensive-care unit.

### Maternal outcomes

Obstetric outcomes for both groups are reported in Table 4. Active obstetric management resulted in the induction of labor in 82.1% of case women, compared to 18.4% of the control group (*P* <0.001). The rate of cesareans during labor did not differ between the groups (*P* = 0.457). In contrast, more scheduled (prelabor) cesareans took place among the case women (12% versus 2%, *P* <0.001). Postpartum hemorrhages were also more frequent in the case group, 25% versus 14.1% (*P* = 0.002), but maternal blood transfusions were not. All severe hemorrhages (blood loss > 1000 mL) occurred in the cholestasis group: six women (17%), four during cesarean deliveries. No significant differences were observed in the rates of coagulopathy, or in prothrombin times and hemoglobin concentrations at the onset of labor.

**Table 4:** Clinical and laboratory characteristics according to the severity of intrahepatic cholestasis of pregnancy (ICP) (N = 140).

	Mild cholestasis 10 ≤ BA ≤ 39 μmol/L N = 83	Moderate cholestasis 40 ≤ BA ≤ 99 μmol/L N = 46	Severe cholestasis BA ≥100 μmol/L N = 11	P Values
Median gestational age at diagnosis (weeks)	36 (33–38)	35 (33–37)	33 (31.5–35)	0.185
Median gestational age at delivery (weeks)	38 (38–38)	38 (37–38)	36 (35–38)	0.009
Respiratory distress syndrome	13 (15.6)	8 (17.4)	4 (36.4)	0.263
Biochemistry at diagnosis				
Bile acid, μmol/L	15 (12–24)	39,5 (21.2–55)	59 (37–100.5)	<0.001
Aspartate transaminase, IU/L	53 (32–84)	95.5 (46.2–170)	220 (100.5–305)	<0.001
Alanine transaminase, IU/L	86 (40.2–148.2)	138 (57–308)	481 (158.5–552.5)	<0.001
Most severe biochemistry				
Bile acid, μmol/L	19 (14–26)	55.5 (43.2–67.5)	131 (115.5–140)	<0.001
Aspartate transaminase, IU/L	54 (31–96)	95.5 (53.2–207.8)	220 (113.5–323)	<0.001
Alanine transaminase, IU/L	79 (38–160)	114 (53–296)	481 (241–611.5)	<0.001
Treatment				
Ursodeoxycholic acid treatment, %	25 (30.1)	30 (83.3)	6 (66.7)	0.252
Term at BA normalization, WG	37 (35–38)	36 (35–38)	32 (32–32)	0.326
Standardization time, days	7 (0.2–12.2)	12.5 (10–28)	16 (16–16)	0.070
Pruritus disappearance, %	20 (31.7)	9 (22.5)	0 (0)	<0.001
Biochemistry at delivery				
Bile acid, μmol/L	13 (8–23)	30 (13–48)	79.5 (45.8–117.2)	<0.001
Aspartate transaminase, IU/L	38 (27–89)	58 (26–94)	119 (33–251)	<0.001
Alanine transaminase, IU/L	59 (30–138)	59 (27–157)	192 (42.5–545.5)	<0.001
Prothrombin time, s	101 (92–112)	100 (99.5–112)	100 (50.9–100)	-
Postpartum hemoglobin, g/dL	12 (11.4–13)	11.7 (10.6–12.3)	11.8 (11.4–12.3)	0.170

### Cholestasis characteristics according to severity

The gestational age at diagnosis (mean, 34.4 ± 2.1 weeks) did not differ significantly by severity, although severe ICP tended to be diagnosed around a week earlier. Hepatic cytolysis was more severe in cases with high BA levels. Hemoglobin levels and prothrombin times did not differ according to the severity of cholestasis. Ursodeoxycholic acid was administered to 43.6% of the cholestasis cases, and the rate of its prescription increased with the severity of the disease. Normalization of the BA most often occurred by 15 days after the onset of treatment. Pruritus disappeared a week after the onset of the treatment in 30% of the women with mild cholestasis and in none of the 11 with severe ICP (Table 3).

## Discussion

Over the 8-year study period, no stillbirths occurred in either group. After adjustment on the confounding factors we found a higher rate of RDS and neonatal morbidity among neonates of the cholestasis group. Mothers with ICP had more postpartum hemorrhages than control women, but did not require more blood transfusions.

The RDS rate was three times higher among neonates of the cholestasis group which is consistent with findings from other studies. Zecca et al, in a case-control study (matching on gestational age) showed a risk of RDS in ICP newborns 2.5 times higher than in control infants (28.6% vs 14%), regardless of BA level [2]. Like others [12], we found that BA was higher in the ICP cases complicated by RDS. Our study also found a significant difference, with more intubation and higher special care and intensive care unit admission rates among case infants. Morbidity in the ICP cases was higher than that in the control population delivered in the same late preterm period [13]. Hypothesis to explain increased neonatal morbidity among case infants include a direct effect of BA on neonatal lung, which could be induce a “bile acid pneumonia” [2, 14]. BA have been found detectable in the bronchoalveolar lavage fluid of case neonates affected by RDS, some authors have speculated that BA inhibits surfactant activity [14]. A meta-analysis suggests that treatment with ursodesoxycholic acid is associated with a decrease in the RDS rate [3]. There were, however, no significant differences in acid-base status or meconium staining during labor, contrary to other studies [15]. The PITCHES trial outcome was to evaluate perinatal outcome in ICP-affected pregnancies of ursodesoxycholic acid versus placebo [16]. Authors funded that treatment with ursodeoxycholic acid does not reduce adverse perinatal outcomes.

The proportion of women with diabetes was higher among ICP cases compared with control, as expected from previous studies [17]. This association could increase stillbirth rates whereas confounding factors are unclear [18]. During ICP, reported stillbirth rates vary between 0.4% and 7% [18,19]. The risk of stillbirth seems to increase after 37 weeks and is rare before 34 weeks. It also increase with BA level [15], when serum bile acids concentrations are of 100 μmol/L or more [20]. Nonetheless, bile acids are not an infallible surveillance marker, and the level can rise abruptly, as shown by the serious accidents reported in this context [10]. Ethnicity (Latino, native American) is also a reported risk factor for stillbirths associated with cholestasis [21], but these ethnicities were not represented in our study. In ICP, stillbirth prevention must be weighed against the long-term consequences of “late preterm” birth [22]. Although the American College of Obstetricians and Gynecologists [8] recommends active management, it does not define an ideal term for childbirth. Two studies advocate that 36 weeks of gestation is the best compromise between the risks of preterm birth and the risk of stillbirth or neonatal death [22–24]. Nonetheless, our results do not support systematic delivery at this late preterm gestational age. Similarly, the Royal College of Obstetrics and Gynaecology does not recommend systematic active management [7]. It concludes that if ICP is associated with stillbirth, which it does not consider statistically proven, the risk is clinically insignificant [11].

Regarding maternal outcomes, the planned cesarean rate was significantly higher in ICP cases. Induction of labor for women with ICP did not increase the emergency cesarean rate [24]. On the other hand, the postpartum hemorrhage rate was higher in ICP cases, probably related to their higher rates of cesarean delivery and of oxytocin-induced labor [25]. We did not, however, observe any differences in the transfusion rates or maternal hemostasis problems. This result is consistent with Brouwers et al. [26].

Our study nonetheless has some limitations. It was a retrospective study with potential bias. Thus, we chose to report and analyze the rates of RDS rather than neonatal unit admission to limit reporting bias.

## Conclusions

Weekly clinical and laboratory monitoring appears essential, although no marker can rule out the onset of in utero fetal death. Treatment with ursodeoxycholic acid does not reduce adverse perinatal outcomes [16]. The risk of RDS, appears to be more frequent in cases of cholestasis regardless of gestational age, and must be taken into account at delivery, even if the birth occurs at term.

## Supporting information

S1 Dataset ICP database scure anonymized. (CSV)
